# Impact of transjugular intrahepatic portosystemic shunt on post-colectomy complications in patients with ulcerative colitis and primary sclerosing cholangitis

**DOI:** 10.1093/gastro/gou085

**Published:** 2014-12-16

**Authors:** Gursimran Kochhar, Udayakumar Navaneethan, Jose Mari Parungao, Jason Hartman, Ranjan Gupta, Rocio Lopez, Arthur J McCullough, Baljiendra Kapoor, Bo Shen

**Affiliations:** ^1^Department of Gastroenterology and Hepatology, The Cleveland Clinic, Cleveland, OH, USA; ^2^Case Western Reserve University School of Medicine, Cleveland, OH, USA; ^3^Department of Vascular and Interventional Radiology, The Cleveland Clinic, Cleveland, OH, USA

**Keywords:** primary sclerosing cholangitis, ulcerative colitis, transjugular intrahepatic portosystemic shunt (TIPS), colectomy

## Abstract

**Objective:** Primary sclerosing cholangitis (PSC) occurs in approximately 5% of patients with ulcerative colitis (UC). The risk of colon cancer is higher in patients undergoing colectomy, who have simultaneous PSC & UC. Our aim was to study the impact, in terms of post-colectomy survival and complications, of transjugular intrahepatic portosystemic shunt (TIPS) before colectomy in these patients.

**Methods:** In this retrospective, case-control study, information was obtained on demographics, disease characteristics, TIPS characteristics, and post-colectomy complications. Nine patients with PSC and UC who underwent TIPS prior to colectomy (the Study group) and 37 patients with PSC and UC who underwent only colectomy without TIPS (the Control group) were included. Either an analysis of variance or the non-parametric Kruskal-Wallis test were used for continuous variables and Fisher’s Exact test or Pearson’s chi-squared test was used for categorical factors.

**Results:** There was no difference in the mean age between the two groups; however patients in the Study group had lower platelet count (*P = *0.005) as well as higher Model for End- Stage Liver disease (MELD) scores (*P < *0.001). Also, patients in the Study group had increased PSC severity as determined by Mayo PSC Risk Scores (1.50 *vs.* 0.20) (*P = *0.001). Total bilirubin levels were higher in the Study group (2.3 *vs.* 0.8 mg/dL) (*P = *0.011). Comparing the post-operative complication rates without adjusting for disease severity, the Study group had more wound infections (*P = *0.034), more wound dehiscence (*P = *0.022), and a higher re-admission rate within 30 days (*P = *0.032); however, the post-operative mortality was not significantly different.

**Conclusion:** Patients with PSC and UC who underwent TIPS prior to colectomy had higher rates of complications; however, this was probably due to the greater severity of cirrhosis and PSC in this population.

## Introduction

Primary sclerosing cholangitis (PSC) is a rare, idiopathic disease with a prevalence of 13.6 per 100 000 persons [1]. It is characterized by inflammation, fibrosis, and strictures of the biliary tree [1–3]. PSC has long been reported as occurring in association with ulcerative colitis (UC) [4–6]. Studies have demonstrated that 44–90% of patients with PSC have underlying UC [1, 7, 8]; however, as few as 5% of patients with UC will simultaneously develop PSC [8]. Some writers have proposed that PSC coinciding with inflammatory bowel disease (IBD) may represent a unique phenotype of IBD, distinct from UC and Crohn’s disease [3, 9].

Patients with PSC often progress to cirrhosis [2]. While the definitive treatment is liver transplantation [3, 10, 11], patients may receive a transjugular intrahepatic portosystemic shunt (TIPS) to manage symptoms such as bleeding esophageal varices, parastomal varices and portal hypertension [12, 13]. Placement of TIPS has been shown to be a minimally invasive and effective means of controlling variceal bleeding, ascites and portal hypertension [12, 14–16].

Patients with PSC and UC also frequently undergo colectomy for the management of UC-related symptoms and complications [17]. Studies have also found that patients with PSC and UC are at high risk for colon cancer [18, 19]. In addition, colectomy has been demonstrated to be protective against recurrence of PSC following liver transplantation [20, 21].

In patients with UC who undergo colectomy, long-term quality of life is considered to be equivalent to that of the normal healthy population [22]; however common post-operative complications of patients with UC undergoing colectomy include wound infection, ileus, bleeding, and the formation of fistulae [23, 24]: major complication rates as high as 27% have been reported [25]. It has been suggested that placement of a TIPS prior to abdominal surgery in cirrhotic patients may improve surgical outcomes [26]; however, there are contradictory studies that found no difference in outcomes [27].

The aim of our study was to determine, in patients who underwent colectomy for the treatment of PSC associated with UC, whether TIPS prior to the colectomy altered their rates of post-operative complications.

## Materials and methods

### Data source

This is a retrospective chart review, approved by our hospital institutional review board (IRB). Data was collected from previously established data base, of all patients undergoing TIPS at our institution from period of 2001 to 2011. This data base was established retrospectively by our department of interventional radiology at Cleveland clinic and includes patient demographic information, indication of TIPS and procedure date.

### Inclusion and exclusion criteria

Patients were included if they underwent colectomy after being diagnosed with PSC and UC at our institution from 2001 to 2011. This was determined using International Classification of Diseases 9^th^ Revision (ICD-9) codes. Patients were excluded from the Study group if they underwent TIPS after colectomy. Patients who met the selection criteria were then divided into two groups: those who had undergone TIPS prior to colectomy (the Study group) and those not having undergone TIPS (the Control group).

### Variables

Demographic information was obtained from the above-mentioned database, including age, gender, race, alcohol use, tobacco use, hepatitis B virus (HBV) and hepatitis C virus (HCV) status, family history of inflammatory bowel disease (IBD), and body mass index (BMI). Information on PSC and UC characteristics was obtained, including duration of PSC, the use of various medical therapies, albumin levels, total bilirubin levels, liver function tests [including aspartate aminotransferase (AST) and alanine aminotransferase (ALT)], alkaline phosphatase (ALP), blood urea nitrogen (BUN), serum creatinine, hemoglobin, platelet counts, international normalized ratio (INR), activated partial thromboplastin time (APTT), and the model for end-stage liver disease score (MELD). The Mayo Risk Score system was used to evaluate the severity of PSC [28].

Information related to colectomy was obtained, including duration of UC at time of colectomy, the indication for colectomy, restoration, and type of ileostomy. Information related to TIPS was obtained, including time from TIPS to colectomy and clinical indication.

### Outcomes of interest

Our primary outcome of interest was post-operative complications following colectomy and whether or not placement of TIPS affected these complications. Complications examined included hypotension, desaturation, bleeding requiring transfusions, re-admission within 30 days, reason for re-admission, ileus, obstruction, wound infection, wound dehiscence, abdominal abscess, pelvic abscess, deep vein thrombosis, proximal vein thrombosis, septicemia, peritonitis, anastomotic leakage, fistula, worsening liver function tests, coagulopathy, pulmonary complications, urinary complications, emergency re-operation, and mortality.

### Statistical analysis

Univariate analysis was performed to assess differences between subjects with and without TIPS. Either analysis of variance or the non-parametric Kruskal-Wallis test were used for continuous variables, and Fisher’s exact test or Pearson’s chi-squared test was used for categorical factors. A *P < *0.05 was considered statistically significant. SAS (version 9.2, The SAS Institute, Cary, NC) was used for all analyses. Data are presented as mean ± standard deviation, median (25th, 75th percentiles) or *n* (%).

## Results

**Figure 1.**
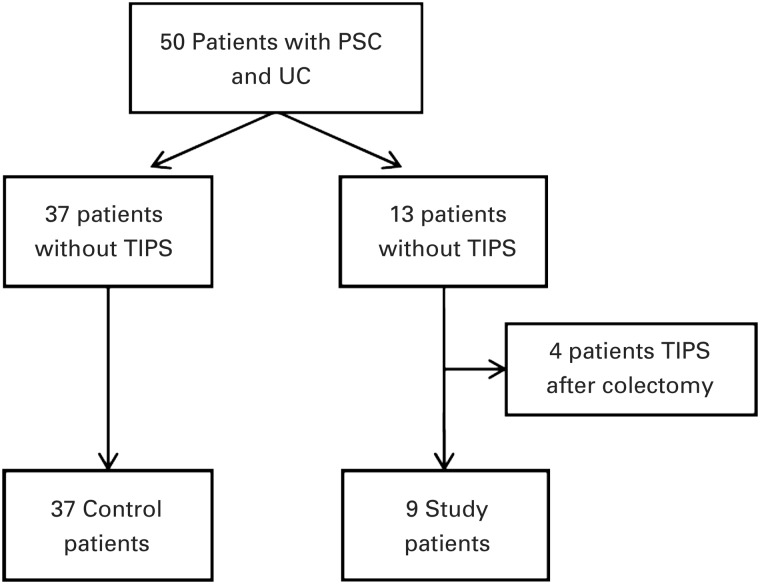
Algorithm of case selection

A total of 50 patients with PSC and UC underwent colectomy. Of these, 13 received a TIPS (the Study group), while 37 did not (the Control group). Four of the 13 TIPS were performed after colectomy and were therefore excluded, leaving 9 patients in the Study group (Figure 1).

### Clinical characteristics

The Study group and the Control group did not significantly differ in terms of age, gender, race, smoking status, alcohol use, HBV status, HCV status, family history of IBD, BMI, or prevalence of obesity (Table 1).

**Table 1.** Demographics and medical history

Factor	n*	No TIPS (*n = *37)	TIPS (*n = *9)	*P*-value
Age at colectomy (years)	45	43.4 ± 14.0	47.8 ± 10.0	0.41
Male	46	27 (73.0)	7 (77.8)	0.77
Race	46			0.99F
Caucasian		35 (94.6)	9 (100.0)	
African-American		2 (5.4)	0 (0.0)	
Alcohol	39	13 (40.6)	0 (0.0)	0.073F
Smoking	40	10 (30.3)	3 (42.9)	0.66F
Human immunodeficiency virus	15	0 (0.0)	0 (0.0)	
Hepatitis B virus	23	1 (5.9)	1 (16.7)	0.46F
Hepatitis C virus	23	0 (0.0)	0 (0.0)	
Family H/O IBD	43	7 (19.4)	2 (28.6)	0.59
Body mass index (kg/m^2^)	36	27.4 ± 6.6	24.9 ± 4.0	0.39
Obesity	36	7 (23.3)	0 (0.0)	0.32F

Values presented as Mean ± SD with ANOVA or *n* (%) with Fisher's Exact test (F) or otherwise Pearson's chi-squared test.

*Partial data are not available in some cases.

TIPS = transjugular intrahepatic portosystemic shunt; IBD = inflammatory bowel disease.

There were a number of significant differences in PSC characteristics between the two groups (Table 2). Patients in the Study group had higher median Mayo Risk Scores (1.5) *vs.* the Control group (0.20) (*P = *0.001). Mean albumin levels were significantly lower in the Study group (2.8 g/dL) *vs.* the Control group (3.8 g/dL) (*P < *0.001). Mean total bilirubin was higher in the Study group (2.3 mg/dL) *vs.* the Control group (0.8 mg/dL) (*P = *0.011). The Study group also had lower hemoglobin (*P < *0.001) and platelet levels (*P = *0.005), higher INR values (*P = *0.026) and MELD scores (*P < *0.001) and longer APTT (*P = *0.045).

**Table 2.** Characteristics of primary sclerosing cholangitis

Factor	n*	No TIPS (*n = *37)	TIPS (*n = *9)	*P*-value
Duration of PSC at colectomy (years)	31	7.0 (2.0, 15.0)	3.5 (2.0, 4.5)	0.31
Severity- Mayo Risk Score	40	0.20 (−0.64, 0.77)	1.5 (0.85, 2.4)	*0.001*
Albumin (g/dL)	40	3.8 ± 0.66	2.8 ± 0.55	*<0.001*
Total bilirubin (mg/dL)	40	0.80 (0.50, 1.3)	2.3 (1.1, 4.8)	*0.011*
Aspartate aminotransferase (U/L)	40	53.0 (27.0, 78.0)	51.0 (36.0, 68.0)	0.97
Alanine aminotransferase (U/L)	39	49.5 (25.5, 83.5)	41.0 (19.0, 118.0)	0.71
Alkaline phosphatase (U/L)	40	227 (123, 387)	410 (249, 497)	0.29
Blood urea nitrogen (mg/dL)	41	11.0 (10.0, 18.0)	15.0 (12.0, 19.0)	0.21
Serum creatinine (mg/dL)	41	0.92 ± 0.34	0.78 ± 0.28	0.3
Hemoglobin (g/dL)	41	12.7 ± 1.9	9.8 ± 0.90	*<0.001*
Platelets (×10^9^/L)	40	329.4 ± 143.6	148.5 ± 95.6	*0.005*
International normalized ratio	37	1.03 ± 0.25	1.3 ± 0.30	*0.026*
Activated partial thromboplastin time(s)	34	30.7 ± 3.5	35.1 ± 8.2	*0.045*
MELD score	37	8.2 ± 2.7	13.8 ± 5.0	*<0.001*

Values presented as Mean ± SD with ANOVA; Median (P25, P75) with Kruskal-Wallis test.

*Partial data are not available in some cases.

TIPS = transjugular intrahepatic portosystemic shunt; MELD = Model for end-stage liver disease

The two groups did not significantly differ in duration of UC at time of colectomy, pre-colectomy treatment, or indications for colectomy; however, patients in the Control group were more likely to undergo restoration (94.6%) than those in the study group (42.9%) (*P < *0.001). Those in the Study group were also more likely to undergo end-type ileostomy, instead of loop-type ileostomy (Table 3).

**Table 3:** Ulcerative colitis and colectomy characteristics

Factor	n*	No TIPS (*n = *37)	TIPS (*n = *9)	*P*-value
Duration of UC at colectomy (years)	40	18.0 (13.0, 33.0)	25.5 (15.0, 30.0)	0.56
Pre-colectomy treatment (non-exclusive)	31			
Anti-inflammatory treatment		18 (66.7)	4 (100.0)	0.30F
Steroids		23 (85.2)	3 (75.0)	0.52F
Immunomodulators		7 (25.9)	2 (50.0)	0.56F
Indication for colectomy (non-exclusive)	44			
Refractory to medications		13 (35.1)	4 (57.1)	0.40F
Dysplasia		19 (51.4)	3 (42.9)	0.99F
Carcinoma		3 (8.1)	0 (0.0)	0.99F
Adenomatous polyps		2 (5.4)	1 (14.3)	0.41F
Restoration	44	35 (94.6)	3 (42.9)	*<0.001*
Type of restoration	38			0.99F
IPAA		33 (94.3)	3 (100.0)	
Ileorectal		2 (5.7)	0 (0.0)	
Ileostomy	45	35 (94.6)	8 (100.0)	0.99F
Type of ileostomy	42			*<0.001*
Loop		33 (94.3)	3 (42.9)	
End		2 (5.7)	4 (57.1)	
Post-operative complications	46	37 (100.0)	8 (88.9)	0.20F
Bleeding	44	6 (16.2)	1 (14.3)	0.9
Ileus	44	8 (21.6)	1 (14.3)	0.66
Obstruction	44	2 (5.4)	0 (0.0)	0.99F
Wound infection	44	4 (10.8)	3 (42.9)	*0.034*
Wound dehiscence	44	0 (0.0)	2 (28.6)	*0.022F*
Abdominal abscess	44	2 (5.4)	2 (28.6)	0.11F
Pelvic abscess	44	6 (16.2)	1 (14.3)	0.9
Deep venous thrombosis	44	0 (0.0)	0 (0.0)	
Portal vein thrombosis	46	1 (2.7)	0 (0.0)	0.99F
Septicemia	44	2 (5.4)	1 (14.3)	0.41F
Peritonitis	44	0 (0.0)	0 (0.0)	
Anastomotic leak	44	4 (10.8)	1 (14.3)	0.99F
Fistula	44	0 (0.0)	1 (14.3)	0.16F
Worsening liver function tests	44	2 (5.4)	0 (0.0)	0.99F
Coagulopathy	44	1 (2.7)	0 (0.0)	0.99F
Pulmonary complications	44	0 (0.0)	1 (14.3)	0.16F
Urinary complications	44	1 (2.7)	0 (0.0)	0.99F
Other complication	37	1 (2.7)	0 (0.0)	0.99F
Blood transfusions requested	42	1 (2.8)	2 (33.3)	*0.049F*
No. of blood transfusion	38			*0.001*
0		33 (94.3)	1 (33.3)	
1		1 (2.9)	1 (33.3)	
4		0 (0.0)	1 (33.3)	
5		1 (2.9)	0 (0.0)	
Hospital stay (days)	44	5.0 (5.0, 8.0)	8.0 (7.0, 23.0)	*0.041*
Re-admission within 30 days	44	7 (18.9)	4 (57.1)	*0.032*
Reason for re-admission (non-exclusive)	11			
Nausea/vomiting		0 (0.0)	1 (25.0)	0.36F
Renal failure		1 (14.3)	0 (0.0)	0.99F
Infection		4 (57.1)	3 (75.0)	0.99F
Wound dehiscence		1 (14.3)	0 (0.0)	0.99F
Bowel obstruction		1 (14.3)	0 (0.0)	0.99F
Emergency re-operation	45	2 (5.4)	0 (0.0)	0.99F
Post-operative mortality	46	0 (0.0)	1 (11.1)	0.20F

Values presented as Median [P25, P75] with Kruskal-Wallis test, or *n* (%) with Fisher's Exact test (F) or otherwise Pearson's chi-squared test.

*Partial data are not available in some cases.

TIPS = transjugular intrahepatic portosystemic shunt; IPAA = ideal pouch-anal anastomosis

Patients underwent placement of a TIPS a median of 2.3 (interquartile 0.68–18.6) months before colectomy. Clinical indications were portal hypertension in six patients, variceal bleeding in one, ascites in one, and “not listed” for one patient.

### Post-operative complications

As illustrated in Table 3, patients who underwent TIPS experienced several complications, with greater frequency than the Control group. The Study group had more intra-operative bleeding—requiring more blood transfusions (*P = *0.049)—more wound infections (*P = *0.034), a higher risk of wound dehiscence (*P = *0.022), and a higher chance of re-admission within 30 days (*P = *0.032). Post-operative mortality did not differ significantly.

## Discussion

To the best of our knowledge, this is the first study of its kind to examine the effect of TIPS prior to colectomy in patients with both PSC and UC. Previous studies have examined the effect of TIPS prior to abdominal surgery in general, with conflicting results; however, these studies examined patients with a variety of diseases undergoing a variety of surgical procedures, while we focused on a specific subset of IBD population (PSC and UC) undergoing one procedure ( Colectomy). [26, 27].

It has been proposed that, in cirrhotic patients, decompression of the portal system through the placement of TIPS prior to abdominal surgery improves surgical outcomes [26]. It has been suggested that, because the TIPS procedure is minimally invasive and successfully reduces portal hypertension—a contra-indication to many abdominal surgeries—it should be used prior to surgery in a two-step process [26]. This claim was based on a study of experiences with seven severely cirrhotic patients, three of whom underwent surgery of the colon [26].

However, other studies have disagreed with these findings. A retrospective examination of the effect of pre-surgery TIPS on patients with cirrhosis undergoing abdominal surgery found that pre-operative TIPS placement did not affect survival, nor did it cause significant differences in post-operative complications [27]. Their study population consisted of 10 patients undergoing colectomy, five undergoing antrectomy, one small-bowel resection, one pancreatectomy, and one nephrectomy while, in the control population of 18 patients, 13 underwent colectomy [26].

These earlier studies led us to hypothesize that TIPS placement prior to colectomy might at best improve outcomes, or at worst offer no significant benefit. It was therefore unexpected that placement of a TIPS was associated with increased intra-operative and post-operative complications.

Our findings suggest that the cause was not the presence of TIPS, but rather that patients in the Study group had more severe cirrhosis, which made both TIPS placement and post-colectomy complications more likely. This agrees with the PSC characteristics of the Study group described above, such as increased Mayo Risk Score, decreased serum albumin, increased total bilirubin, increased INR, increased APTT, and increased MELD score, all of which suggest more severe cirrhosis. This suggests that patients with severe disease, who undergo TIPS, are still at risk of worse outcomes than patients with mild disease, irrespective of the presence or absence of TIPS.

The increased INR and APTT explain the increased intra-operative bleeding requiring blood transfusions. The increased rate of wound infections seen in the Study group may also be the result of more severe cirrhosis. Various studies have shown that patients with cirrhosis are likely to acquire secondary infections while hospitalized [29, 30]; it is thought that patients with cirrhosis have impaired immune function [31]. Studies have also suggested that patients with cirrhosis produce less glutathione, which increases their risk of infection [32, 33]. It is also possible that those in the Study group were taking higher doses of immunomodulators; this could not be ascertained, as we only looked at use of immunomodulators as a categorical variable.

Wound infection has been shown to be a major risk factor in the development of wound dehiscence following abdominal surgery, through the increased presence of neutrophils and matrix metalloproteinases [34]. This may explain the increased rate of wound dehiscence observed in our Study group. The higher rates of wound infection and dehiscence offer an explanation for the increased re-admissions within 30 days.

This study is limited by its retrospective nature and the use of databases, which may have introduced further confounding factors such as incorrect coding. We were also limited by the fact that we were examining a rare disease: out of our entire database, we were only able to find nine patients to include in the Study group, which may not have been large enough to provide sufficient power to detect all complications. Data on liver transplantation and dosing regimens of immunosuppressive medications might have been useful but were not available. In addition, this is a single-institutional study, which limits the general applicability of our findings.

In conclusion, our findings suggest that patients who underwent TIPS placement had more severe cirrhosis, which led to an increase in intra-operative and post-operative complications. But what remains unclear is whether TIPS placement may have exacerbated or diminished these complications. Based on our initial findings, prospective tracking of patients with PSC and UC undergoing colectomy following TIPS may be warranted.

*Conflict of interest statement*: none declared.

## References

[1] Bambha K Kim WR Talwalkar J Incidence, clinical spectrum, and outcomes of primary sclerosing cholangit in a United States community. Gastroenterology 2003;125:1364–9. 14598252 10.1016/j.gastro.2003.07.011

[2] Wiesner RH Grambsch PM Dickson ER Primary sclerosing cholangitis: natural history, prognostic factors and survival analysis. Hepatology 1989;10:430–6. 2777204 10.1002/hep.1840100406

[3] Karlsen TH Boberg KM Update on primary sclerosing cholangitis. J Hepatol 2013;59:571–82. 23603668 10.1016/j.jhep.2013.03.015

[4] Atkinson AJ Carroll WW Sclerosing cholangitis. Association with regional enteritis. JAMA 1964;188:183–4. 14107248

[5] Schaeffer DF Win LL Hafezi-Bakhtiari S The phenotypic expression of inflammatory bowel disease in patients with primary sclerosing cholangitis differs in the distribution of colitis. Dig Dis Sci 2013;58:2608–14. 23670229 10.1007/s10620-013-2697-7

[6] Smith MP Loe RH Sclerosing cholangitis; review of recent case reports and associated diseases and four new cases. Am J Surg 1965;110:239–46. 14313190 10.1016/0002-9610(65)90018-8

[7] Escorsell A Pares A Rodes J Epidemiology of primary sclerosing cholangitis in Spain. Spanish Association for the Study of the Liver. J Hepatol 1994;21:787–91. 7890895 10.1016/s0168-8278(94)80240-8

[8] Tung BY Brentnall T Kowdley KV Diagnosis and prevalence of ulcerative colitis in patients with sclerosing cholangitis (abstract). Hepatology 1996;24:169A.8707258

[9] Loftus EV Jr Harewood GC Loftus CG PSC-IBD: a unique form of inflammatory bowel disease associated with primary sclerosing cholangitis. Gut 2005;54:91–6. 15591511 10.1136/gut.2004.046615 PMC1774346

[10] Langnas AN Grazi GL Stratta RJ Primary sclerosing cholangitis: the emerging role for liver transplantation. Am J Gastroenterol 1990;85:1136–41. 2167607

[11] McEntee G Wiesner RH Rosen C A comparative study of patients undergoing liver transplantation for primary sclerosing cholangitis and primary biliary cirrhosis. Transplant Proc 1991;23:1563–4. 1989291

[12] Pennick MO Artioukh DY Management of parastomal varices: who re-bleeds and who does not? A systematic review of the literature. Tech Coloproctol 2013;17:163–70. 23152077 10.1007/s10151-012-0922-6

[13] Kawamata H Kumazaki T Kanazawa H Transjugular intrahepatic portosystemic shunt in a patient with cavernomatous portal vein occlusion. Cardiovasc Intervent Radiol 2000;23:145–9. 10795842 10.1007/s002709910030

[14] Burroughs AK Vangeli M Transjugular intrahepatic portosystemic shunt *vs.* endoscopic therapy: randomized trials for secondary prophylaxis of variceal bleeding: an updated meta-analysis. Scand J Gastroenterol 2002;37:249–52. 11916185 10.1080/003655202317284138

[15] Salerno F Camma C Enea M Transjugular intrahepatic portosystemic shunt for refractory ascites: a meta-analysis of individual patient data. Gastroenterology 2007;133:825–34. 17678653 10.1053/j.gastro.2007.06.020

[16] Garcia-Tsao G The transjugular intrahepatic portosystemic shunt for the management of cirrhotic refractory ascites. Nat Clin Pract Gastroenterol Hepatol 2006;3:380–9. 16819501 10.1038/ncpgasthep0523

[17] Cima RR Pemberton JH Medical and surgical management of chronic ulcerative colitis. Arch Surg 2005;140:300–10. 15781797 10.1001/archsurg.140.3.300

[18] Venkatesh PG Jegadeesan R Gutierrez NG Natural history of low grade dysplasia in patients with primary sclerosing cholangitis and ulcerative colitis. J Crohns Colitis 2013;7:968–73. 23433613 10.1016/j.crohns.2013.02.002

[19] Knechtle SJ D'Alessandro AM Harms BA Relationships between sclerosing cholangitis, inflammatory bowel disease, and cancer in patients undergoing liver transplantation. Surgery 1995;118: 615–20. 7570313 10.1016/s0039-6060(05)80026-1

[20] Vera A Moledina S Gunson B Risk factors for recurrence of primary sclerosing cholangitis of liver allograft. Lancet 2002;360:1943–4. 12493264 10.1016/S0140-6736(02)11861-7

[21] Alabraba E Nightingale P Gunson B A re-evaluation of the risk factors for the recurrence of primary sclerosing cholangitis in liver allografts. Liver Transpl 2009;15:330–40. 19243003 10.1002/lt.21679

[22] Heikens JT de Vries J van Laarhoven CJ Quality of life, health-related quality of life and health status in patients having restorative proctocolectomy with ileal pouch-anal anastomosis for ulcerative colitis: a systematic review. Colorectal Dis 2012;14:536–44. 21176062 10.1111/j.1463-1318.2010.02538.x

[23] Marcello PW Milsom JW Wong SK Laparoscopic total colectomy for acute colitis: a case-control study. Dis Colon Rectum 2001;44:1441–5. 11598472 10.1007/BF02234595

[24] Marceau C Alves A Ouaissi M Laparoscopic subtotal colectomy for acute or severe colitis complicating inflammatory bowel disease: a case-matched study in 88 patients. Surgery 2007;141:640–4. 17462464 10.1016/j.surg.2006.12.012

[25] Coakley BA Telem D Nguyen S Prolonged pre-operative hospitalization correlates with worse outcomes after colectomy for acute fulminant ulcerative colitis. Surgery 2013;153:242–8. 23062652 10.1016/j.surg.2012.08.002

[26] Azoulay D Buabse F Damiano I Neoadjuvant transjugular intrahepatic portosystemic shunt: a solution for extrahepatic abdominal operation in cirrhotic patients with severe portal hypertension. J Am Coll Surg 2001;193:46–51. 11442253 10.1016/s1072-7515(01)00911-5

[27] Vinet E Perreault P Bouchard L Transjugular intrahepatic portosystemic shunt before abdominal surgery in cirrhotic patients: a retrospective, comparative study. Can J Gastroenterol 2006;20:401–4. 16779457 10.1155/2006/245082 PMC2659922

[28] Kim WR Therneau TM Wiesner RH A revised natural history model for primary sclerosing cholangitis. Mayo Clin Proc 2000;75:688–94. 10907383 10.4065/75.7.688

[29] Bajaj JS O'Leary JG Reddy KR Second infections independently increase mortality in hospitalized patients with cirrhosis: the North American consortium for the study of end-stage liver disease (NACSELD) experience. Hepatology 2012;56:2328–35. 22806618 10.1002/hep.25947 PMC3492528

[30] Park HJ Lee YM Bang KM Clinical significance of Staphylococcus aureus bacteremia in patients with liver cirrhosis. Eur J Clin Microbiol Infect Dis 2012;31:3309–16. 22833245 10.1007/s10096-012-1697-4

[31] Taneja SK Dhiman RK Prevention and management of bacterial infections in cirrhosis. Int J Hepatol 2011;2011:784540. 22229097 10.4061/2011/784540 PMC3168849

[32] Czeczot H Scibior D Skrzycki M Glutathione and GSH-dependent enzymes in patients with liver cirrhosis and hepatocellular carcinoma. Acta Biochim Pol 2006;53:237–42. 16404476

[33] Morris D Khurasany M Nguyen T Glutathione and infection. Biochim Biophys Acta 2013;1830:3329–49. 23089304 10.1016/j.bbagen.2012.10.012

[34] van Ramshorst GH Nieuwenhuizen J Hop WC Abdominal wound dehiscence in adults: development and validation of a risk model. World J Surg 2010;34:20–7. 19898894 10.1007/s00268-009-0277-y PMC2795859

